# User Experience Sensor for Man–Machine Interaction Modeled as an Analogy to the Tower of Hanoi

**DOI:** 10.3390/s20154074

**Published:** 2020-07-22

**Authors:** Arkadiusz Gardecki, Michal Podpora, Ryszard Beniak, Bartlomiej Klin, Sławomir Pochwała

**Affiliations:** 1 Faculty of Electrical Engineering, Automatic Control and Informatics, Opole University of Technology, 45-758 Opole, Poland; a.gardecki@po.edu.pl (A.G.); r.beniak@po.edu.pl (R.B.); 2 Weegree Sp. z o.o. S.K., 45-018 Opole, Poland; b.klin@weegree.com; 3 Faculty of Mechanical Engineering, Opole University of Technology, 45-271 Opole, Poland; s.pochwala@po.edu.pl

**Keywords:** AI system, user experience testing, process control, human supported systems, experience-centered paradigm

## Abstract

This paper presents a novel user experience optimization concept and method, named User Experience Sensor, applied within the Hybrid Intelligence System (HINT). The HINT system, defined as a combination of an extensive AI system and the possibility of attaching a human expert, is designed to be used by relational agents, which may have a physical form, such as a robot, a kiosk, be embodied in an avatar, or may also exist as only software. The proposed method focuses on automatic process evaluation as a common sensor for optimization of the user experience for every process stage and the indicator for human-expert automatic session activation. This functionality is realized by the User Experience Sensor, which constitutes one of main elements of the self-optimizing interaction system. The authors present the optimization mechanism of the HINT system as an analogy to the process of building a Tower of Hanoi. The proposed sensor evaluates the user experience and measures the user/employee efficiency at every stage of a given process, offering the user to choose other forms of information, interaction, or expert support. The designed HINT system is able to learn and self-optimize, making the entire process more intuitive and easy for each and every user individually. The HINT system with the proposed sensor, implemented in a window assembly facility, successfully reduced assembly time, increased employees’ satisfaction, and assembly quality. The proposed approach can be implemented in numerous man–machine interaction applications.

## 1. Introduction

The meaning of the User Experience (UX) term, as proposed in [1], was primarily meant to include all aspects of users’ interaction with a particular product. Nowadays, the UX is defined within an ISO standard [2] and refers mainly to the consequences of the distinction between “use” and “anticipated use” [3]. The term UX is heavily used in application and interface design. Therefore, the meaning of UX shifts towards User-Centered Design or even purely Graphical User Interface, which is an oversimplification.

The general Human-Computer Interaction (HCI) needs a wider definition of UX than just a decent Graphical User Interface. Thus, more aspects are taken into consideration, including: usability [3], effectiveness, efficiency, and satisfaction [4]. The user satisfaction is described [5] as a result of the following five aspects: aesthetics, likeability, emotion, expectation and usability.

The user experience in human–machine interaction seems to be very similar to the UX in HCI, but it is not. When interacting with a computer system, e.g., via a web browser, people accept/tolerate some typical problems (including answer delays or too-general search results). However, when interacting with a robot/humanoid, people expect fluent and expressive interaction. Therefore, the efficiency and organization of each of the “building blocks” (microservices, subsystems, brokers) of the information path should be carefully planned and optimized. This article is the result of a new approach to this issue—the authors propose not only the conception of individual tasks as “building blocks”, but also an additional supervising software layer that is able to evaluate, manage and optimize the execution of individual blocks as well as the whole interaction.

### 1.1. Introduction to the Hybrid Intelligence System (HINT)

Artificial Intelligence (AI) and natural language conversational systems (so-called chatbots) are a very popular and promising branch of the Human–Machine Interfaces (HMI) sector. Despite significant progress [6] in the field of human-machine communication (including Google Speech [7], updated Siri [8] for Apple devices (VI 2017), as well as dedicated devices (including: Amazon Echo, Google Home) and noticeable acceleration [9] in the subject of artificial intelligence (also due to the popularization of deep architectures [10]), AI systems still differ significantly from popular science visions and ideas. For this reason, in order to get a system capable of remarkable quality of man–machine communication, especially in non-standard or unexpected situations/scenarios, the authors have decided to combine following three approaches:

- an AI pre-programmed with specific facts and topics within a predefined domain of expert knowledge,
- the ability to learn/supplement the knowledge by inserting new facts and relations,
- an assistance of a human expert in case of lack of knowledge or any other emergency situation.

The term Avatar can be perceived as (1) modeling human interlocutors in the virtual environment [11,12] (e.g., virtual assistants) or (2) as the presence of (additional) human resource within the virtual environment [12,13,14]. In this article, the authors use the latter understanding of the Avatar concept.

### 1.2. The Research Objective

The main idea behind the HINT project was to build a hybrid system containing two key parts: a basic automatic natural language conversational system, and an external service offering the possibility of attaching a human expert to the system, hereinafter referred to as the Avatar. The system is primarily intended to enable voice communication and (complementarily) other forms of communication. Since the project was funded under enterprise innovation support funds, the development phase focused specifically on applications in production processes. However, the area of application actually goes far beyond this scope.

The main research objective behind the proposed conception is the verification whether the use of such system (containing a module that enables optimization of the production process) allows to reduce the time of process execution while ensuring its correctness.

### 1.3. Advantages and Limitations of the Proposed System

Implementing the above-mentioned HINT system including the proposed User Experience Sensor results in following benefits:

1. The system, its behaviour and interface is intuitive enough to be used by untrained employees.
2. The system supports quick employee training and efficient problem solving [15].
3. Improvement of the quality of work [16].
4. Relieving highly qualified employees from time-consuming activities (such as teaching beginners) [17] (see part 4, chapter 13).
5. Causing an employee not to feel watched/supervised by another person, which is a considerable discomfort for some people [18].
6. The system allows employees to make decisions independently within certain limits. The employee can choose the type and form of information that suits him best, with particular groups of employees choosing different forms of providing virtually the same information [19].
7. The system is able to analyze the effectiveness of the information provided, and to check if another form of information would be better for a given employee (as a result of: employee’s request to change the form, numerous changes of the information form or the Avatar’s suggestion after interaction), and thus modifying the employee’s preferences to such preferences that increase efficiency [16].

Disadvantages and limitations:

1. The need to build an appropriate system.
2. The need for a proper space to mount the interaction hardware (screen, microphone, camera, etc.).
3. The need to include the documentation and manuals into the system.
4. The system requires good availability of an expert with high knowledge and special predispositions to fit the role of an Avatar.

## 2. Materials and Methods

The HINT system consists of both hardware and software layers and involves the use of many advanced external services. The challenge was to propose a system offering stable execution in the context of the integration of numerous technologies used. The following are the assumptions of the HINT system and the technologies used.

### 2.1. The Conception of the Proposed Hybrid Intelligence System (HINT)

The proposed HINT system was meant to meet the market need regarding the availability (lack) of qualified employees. Many employers in their business models assume the possibility of massive staff rotation, including employees of various nationalities, as well as foreign company branches, with different levels of language competence. The HINT system is designed to support the production process for this group of employees, mainly in the areas of:

- communication with the system in various languages,
- process support described by stages (e.g., custom manufacturing)—products are manufactured based on customer orders,
- production stage control,
- in problem cases, by providing access to a human-expert (Avatar).

In the authors’ assumption, this system should be as automated as possible and every instance of a contact with Avatars should lead to system optimization, so that similar problem situations could be automatically handled over time.

The structure of the human work supervision system is also intended to solve the problem of detecting the moment of connection with the Avatar, by analyzing the stages and times of the process. It is treated by the authors as the experience sensor that detects the need for the Avatar to support the interaction. This detection is possible because such a system can automatically (autonomously) analyze the behavior (experience) of the employee regarding the process. In this approach, the Sensor system consists of a telemetry system and a signal analysis system.

**Figure 1:**
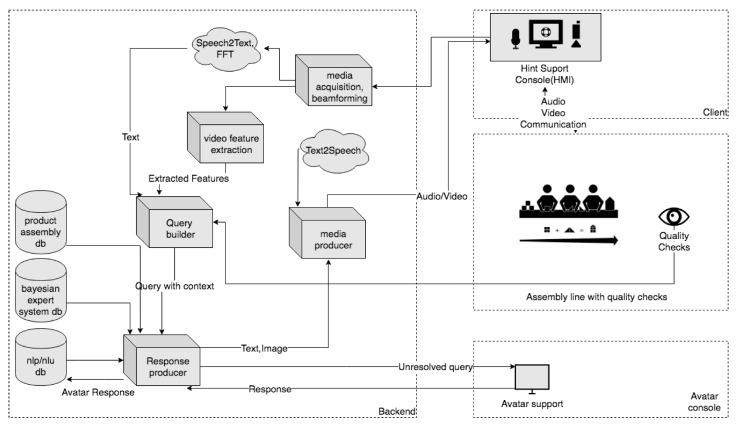
Main components diagram of the Hybrid Intelligence System (HINT) system.

Figure 1 depicts the main modules of the HINT system and their internal relations.

The HINT system is modular, which makes it possible to provide wide integration possibilities and leaves proper flexibility for implementing additional functionalities and improvements during product development.

From the macroscopic (low level of detail) point of view, the main elements of the system are:

- backend—as a unit that processes audio and video streams into inputs of other components, generates a response to the HMI and controls the work of components dependent on it,
- Avatar console—as an interface unit for an expert supporting/supervising the process/processes,
- client—as a unit that (1) acquires physical data and forwards it to a backend, and (2) is an output interface for the response generated by backend.

The HINT system integrates modern techniques of Natural Language Processing (NLP) based on Machine Learning and Artificial Intelligence (the exact service being used in the system is the Speech-to-Text (STT) cloud service, provided by Google [20]). This allows the processing of the natural speech by an NLP/NLU module (NLU—Natural Language Understanding) and mapping the interlocutor’s intentions to predefined actions within/by the HINT hybrid intelligence engine. As a result, an appropriate response for a user is generated or the Avatar Console support session is initiated if the certainty of the answer given does not exceed a specific threshold. For this purpose, the Google DialogFlow [21] is being used in the current version of the prototype and product.

The hardware of the HMI, is an output and input device, typically a terminal equipped with a monitor, speakers, a microphone, and a camera, yet the interaction hardware could be also (depending on the application) a humanoid robot, e.g., Pepper [22] or Sanbot [23]. Using e.g., a built-in screen, the HMI can present specific information provided by the current scenarios running in a hybrid intelligence engine, supported online by the supervisor/expert via the avatar console. The HMI is also intended to perform verbal and non-verbal interactions, e.g., by using the context menus displayed on the built-in screen, as well as a touch screen.

Assuming system integration with the components of the production line responsible for quality assurance, the HINT system, by supplementing contextual data with information obtained from the above components, effectively supervises and updates instructions to optimize the process scheduled/intended on the production line and thus increase the quality.

### 2.2. Avatar

In the HINT system, in addition to traditional interaction channels, there is a possibility to engage with a human expert/specialist acting as an additional source of process knowledge. This option is called by the authors of this paper an “Avatar” and it is the basis for recognizing this system as a hybrid—a combination of automatic and human resources. The Avatar’s console, as well as the very idea of combining artificial intelligence with human resources, is the basic component of the proposed system and is what creates its strength. The operator’s primary task is to ensure uninterrupted and flawless conversation between an interlocutor and a specific interface of the HINT system (e.g., a touchscreen and NLP implemented or a humanoid robot), by actively assisting the system in conversation when the built-in artificial intelligence engine is insufficient.

The adopted direction of system design and development can be significantly beneficial e.g., for people with physical disabilities/limitations and/or for remote work. It is also a technical challenge while the presence of an Avatar can be explicit or masked within the information path of the system.

Considering the role played by a human specialist (Avatar) in the proposed man-machine interaction process, the following aspects of the system (resulting from his participation and from the Avatar’s console interface functionality) should be indicated:

1. flawless switching between autonomous conversation system (human – AI) and assisted conversation mode (human–specialist) giving the impression of an unlimited artificial intelligence technology to the interlocutor,
2. support of the system’s knowledge by the operator’s knowledge, by supplementing it by adding new facts and relations to the system,
3. solving problematic situations in a manner typical of a human being, as opposite to a manner typical of information systems,
4. and others not mentioned here.

In terms of existing technologies, the proposed solution (as well as the concept of the human-expert/Avatar in the system) can be seen in following ways:

- from the human interlocutor’s point of view: extending the intelligence and knowledge of the robot (seems to be similar to attempts to deceive the Turing Test by helping the AI),
- from the robot’s point of view: supporting the system in the situation of absence of information necessary to continue the conversation and supplementing the system’s knowledge.

### 2.3. Key Technologies

To perform an effective machine–human dialogue, it is necessary to transform the analogue signal of human speech (sound wave) into the text form being the input for the chatbot/AI engine (response generator). Moreover, it is important to eliminate or reduce the impact of any interference affecting the data.

The following aspects and approaches prove to be helpful:

- Active Beamforming [24]—gives the possibility to filter the acquired sound basing on the source location in two- or three-dimensional space, using a microphone matrix. Using Active Beamforming within the vision system [25] feedback loop [26] provides additional information on the location of the interlocutor, and the beamforming subsystem can be reconfigured to target the acquisition coordinates to a specific area/direction,
- Speech-to-text conversion—a spectrogram of a specified time segment of the recorded sound sample can be analyzed based on large recursive neural networks [27,28]. In order to improve the recognition efficiency of the most frequently issued commands, the system has been expanded with an additional context containing quantitative frequency data generated by FFT for each of the system users and a subsystem of enhancing important signal features [29],
- Natural language processing in the above-described system is designed to convert text into structured data and determine the interlocutor’s intentions along with the preparation of data for the expert network,
- Expert Bayesian Networks [30]—for making decisions in the conditions of incompleteness and uncertainty of input data based on the structured context provided by NLP as well as subsystems providing only context data (video subsystem, quality subsystem).

The information on the basis of which the decision will be made creates a knowledge base. A distinction should be made here between the knowledge base for the NLP system (NLP Base), its task is to structure natural speech to values accepted by the system, and the knowledge base for the expert network system (Bayes Base). In addition, the system has the process instruction manual implemented (Process Base), which gives the possibility to supervise and properly support the interlocutor in the production process.

### 2.4. Operation Mode 1—(Semi-) Unattended Autonomous Mode with Optional Avatar Support

**Figure 2:**
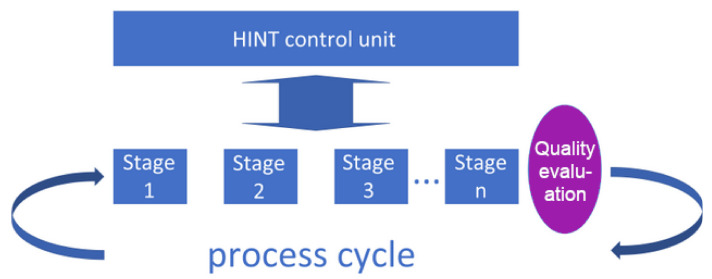
Process mode with verification at the end of the cycle (lightly supervised).

The HINT system includes two main modes of processing/handling system interaction with a user: (1) a mode with a small participation of verification systems (lightly supervised) and (2) a mode with regular participation of such systems (heavily supervised, described in the subsection below). In the first case (1), visualized in Figure 2, the hybrid intelligence scenario has access to a dedicated knowledge base (Bayes and Process Base) on the basis of which it makes decisions and from which it takes the knowledge transmitted/presented to the interlocutor in accordance with the stage of the process. This mode is intended for less critical processes, where due to cost or organizational reasons, the correctness verification occurs only after the end of the cycle or in such moments when the verification would require human intervention (see Figure 2).

For a lightly supervised process, the sensor that determines the poor preparation of the employee (the need to call the Avatar) is activated when the instructions for the stages are too often repeated for any stage of the process or when the time of lightly supervised process deviates far from the assumed times of execution of the currently implemented stage of the process.

At the end of the cycle, compliance of the stage patterns with the actual state is assessed and action can be taken if the assessment is too low. During the stages, however, it is possible to control the parameters of selected subprocesses (comprising the stages) and take action based on them, e.g., the decision to automatically initiate and join the Avatar stage (call a human-expert to support the stage remotely) or to detect an activity indicating the lack of readability or correctness of the stage or instructions, which may also reflect in the UX assessment. The UX assessment can be used for optimizing the interaction process within every stage in order to choose the best interaction means and channels. The concept proposed by the authors, an analogy to the Tower of Hanoi, is presented in Section 2.7. The proposed algorithm enables such activities based on observation of a stage, as well as on the differences between the result and the reference process. The sensors in this case provide the necessary information about the process, which the subsystem obtained by the telemetry sends to the analysis system. The sensors used differ from one stage to another and we deliberately do not define them here in detail, focusing only on the analysis of the information they provide.

### 2.5. Operation Mode 2—Auto-Corrected Autonomous Mode with Optional Avatar Support

**Figure 3:**
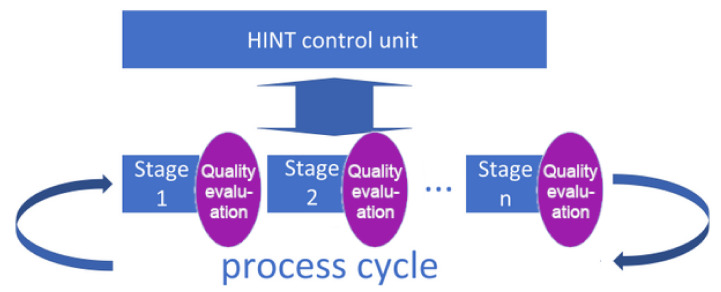
Process mode with stage verification during the cycle (heavily supervised).

The mode of operation with regular participation of verification systems (Figure 3) allows earlier and more reliable detection of deviations from the assumptions of a result or duration of a specific stage within the process. However, it requires additional effort. There are also processes containing stages in which this verification is almost impossible in an automatic system and requires the participation of a human factor. In this case, it is also worth using the conception described in Section 2.7 to assess the course of the stage and its impact on the UX or to decide if the Avatar session should be initiated.

The whole cycle of a single utterance within a conversation is evaluated regarding certainty, and in the case of a low rating, the final decision on the content of the answer can be reviewed and/or made by the Avatar (a human-expert, supporting the system during interactions):

- the text of the answer is not modified, but confirmed by the Avatar, causing the system to raise the confidence parameter (this particular answer will be used more frequently in similar future situations, possibly without the need to disrupt the Avatar),
- the text of the answer is modified by the Avatar, and the system will use a few question–answer pairs to re-learn this particular situation, and thus to improve the knowledge base (Bayes- and Process-bases) as well as the NLP engine (NLP bases).

Due to the built-in communication module, it is also possible to include additional technologies and/or people within the communication path by using e.g., Viber or VoIP (Voice over IP) telephony.

### 2.6. The Reasons Causing the Initialization of Connection with an Avatar

Switching the information path (conversation) from automatic mode to the Avatar mode should be available both on demand and when initiated by the HINT system based on the premises of the process stages control mechanism.

The operation of the User Experience Sensor (designed inter alia for detecting the need to attach an Avatar) for the heavily supervised and lightly supervised systems are similar, except that in the case of a heavily supervised system the correctness of the stage performance is additionally checked after each stage.

The following is a map of behavior that can cause to switch the conversation to the Avatar-supported mode within the HINT system:

1. Upon the Avatar’s request
  - At any time, the Avatar that is observing the course of the conversation can take over the conversation, thus replacing the AI engine output by typing the answer/text directly with a keyboard. This option is used rarely, only if a particular customer needs to be served in a perfect way (completely trouble-free, without delays, without missed answers, and even without universal/fuzzy answers).
  - Avatar can take over the interaction of any agent when he/she needs fast communication with people in the case of an emergency (e.g., the camera of the robot captured an event like a fire or a pickpocket and the Avatar wants to warn the robot’s interlocutor or to ask him for a specific action)
  - Avatar can take over the interaction of an agent, if the values of conversation quality parameters (STT duration or confidence level -or- conversation topic identification) or values of technical parameters (e.g., increased transmission latency to/from cloud services) are relatively low—the Avatar can improve the overall interaction quality by taking over the conversation
2. Initiated by NLP engine of the HINT system
  - If the NLP or SpeechToText engine of the HINT system does not correctly recognize the topic or parameters of a human speech/text or the expert network explicitly recommends getting help, it may suggest/request Avatar assistance
  - If a given interaction path does not hold one/some of the technical parameters or does not provide the availability of a required service (including SpeechToText service delays, unacceptable ping or jitter, no internet connection, etc.)
3. Initiated by the HINT knowledge base module broker
  - If the NLP engine correctly recognizes the topic, subject, intention, history of the conversation but the knowledge base lacks a given value/topic/article/subject, the system may request Avatar assistance
  - In case the NLP engine correctly recognizes the topic, subject, intention, history of the conversation, but there are additional elements that interfere with the interpretation (e.g., the verbs: connect, fix, break), whose presence reduces the score value of some indicators of the quality of substantive conversation.
4. Initiated by the HINT process stage control system
  - If the stage control system described in Section 2.7 provides premises for activating the Avatar connection option
  - Or, if other subsystems of the HINT system have security incidents implemented (e.g., movement at night, smoke or fire), the system may trigger various alarm actions, one of which is activating the remote connection with the Avatar.

The above-mentioned proposals of criteria for switching the course of conversation from autonomous mode to the Avatar mode should be understood as suggestions, prompting to consider appropriate use of cases, not as a closed set that cannot be exceeded.

### 2.7. The Challenge

The authors propose automatic optimization of the efficiency of the man–machine interaction process, taking into account stage patterns and the use of stage information, to automatically activate the option of attaching a human-expert (Avatar). The same mechanism can be a hint for optimizing a stage and give feedback information indicating the area affecting UX evaluation.

Consider the process implemented with the support of: (1) a learning system with indirect supervision over the implementation process or (2) a learning system with direct supervision over the process, or (3) a system based on (1) or (2) with the possibility of contacting with the human-expert supervising the system, having in-depth knowledge of the processes occurring in the system. Each such process consists of supervised or unsupervised stages, and at each stage of such a process all of the information available in the system can be used. However, the manner of processing and presenting information as well as the numerous repeated playback (if needed) affect the efficiency of process execution, i.e., the overall time needed to complete all stages of a process implementation.

Any process implemented in such a system can be represented by the process of building the Tower of Hanoi. Some assumptions are necessary for such representation to be reliable and allow for an autonomous analysis of a completed or currently implemented process:

- first of all, it is assumed that the diameters of the rings placed on the tower will be determined and adjusted to the number of stages of the entire analyzed process.
- second, to clarify the analysis, it is assumed that the ring corresponding to the first stage of the process have larger diameter and that the rings will gradually decrease their diameters, so that the rings corresponding to the initial stages of the process have larger diameters than those corresponding to the final stages.
- third, the height of individual rings will depend on and result from the duration of performing a given stage of the process.
- fourth, the colors used in the rings will reflect the system user’s actions possible in the system—various information paths (e.g., passing information using speech, text, video or by using a default response).

**Figure 4:**
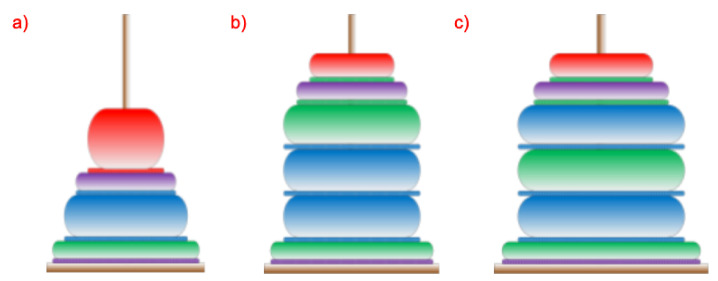
Sample Towers of Hanoi and their corresponding descriptions: (**a**) tower in which no stage had to be repeated, (**b**) tower with one stage repeated using two interaction methods, (**c**) tower with repeatedly changed form of obtaining information.

The operation of the system is sequential and cyclic. Each stage or section of the stage consists of user interaction and system response. Due to the possible types of interactions with the system including: text/touch, speech, avatar session or waiting for a default response (for a predefined time), four colors have been proposed and distinguished at each stage: green (speech-based communication), blue (touchscreen-based interaction), red (avatar-assisted interaction, i.e., with help of an expert) and purple (waiting for a default response). After every interaction with the system, the system is supposed to respond. The answer can be given by: video (blue), text and a speech message (green), avatar session call, or a default response, which is one of the three previously mentioned. The default response is the one which, from the system point of view, is the most efficient on the given stage of the process/interaction. Of course, any type of activity can be repeated many times at every stage. However, this results in a longer time needed to complete the required action/stage. During the realization of each stage, various system activities are required. These activities include system’s interaction with the human-operator and thus the interaction as a command or question being spoken in a voice by the human should imply the following system’s activities: Speech to Text (STT) conversion, sending text to the chatbot/AI subsystem, finding the right/corresponding action and/or answer needed, and providing an answer. Answers can be made by playing a film, displaying a photo or a text, or by reading a message using a Text-to-Speech (TTS) generator. A system user (human employee) can choose their preferred method of presenting information. Each colored ring corresponding to a process step has an associated colored ring for answering or providing information from the system to the user. An example of the proposed structure of Hanoi towers is shown in Figure 4. For simplicity, let us assume that the overall process consists of only four stages.

Exemplary information path of the system’s subroutines include: conversion of speech into text (green), sending a text-based query to the database (blue), the response from the database (orange) or the answer given by the Avatar (red). The possible repetitions of rings of the same diameter, as well as the order of occurrence of the colors indicate that the user has had numerous attempts within a specific stage. Let us also keep in mind that the user’s selected action can be broken down into many complex system subroutines. After the analysis of various interaction forms of a particular query sent to the system, as well as the response from the system (preceded by the required information processing), it can be concluded that in typical situations, the time required for system operation is significantly shorter than the operation time of a human user at a given process stage. Comparison of the shape and colors of the towers for various system users allows to analyze their work efficiency and, through the analysis of the occurrence of colors, to learn the user’s preferences in choosing the system operation mode. Therefore, analyzing these preferences for many users gives the possibility to optimize the operation of the system. The superior system, by analyzing the parameters (presented as shapes and colors of the towers), gives the possibility to optimize system preferences in the selection of the best-preferred system operation on a given query.

Such a choice is possible because the best forms of a question chosen by the users and the forms of answers related to that question will gradually displace other possibilities. In addition, applying the best forms of interaction with the system will reduce the number of erroneous actions and, consequently, the number of repetitions and the overall time of performance of a given stage. Such optimization is particularly beneficial in the case of the lightly supervised activities of a person using the system. With regard to heavily supervised actions, involving a thorough check of the effects of a particular stage, the system’s functionality optimization is also possible, but the optimization is limited to choosing the optimal form of information transfer with the user.

#### The System Operation Strategy Matched with the Tower of Hanoi Structure and the Impact of the Choice of System Processes on the Duration of the Stage

**Figure 5:**
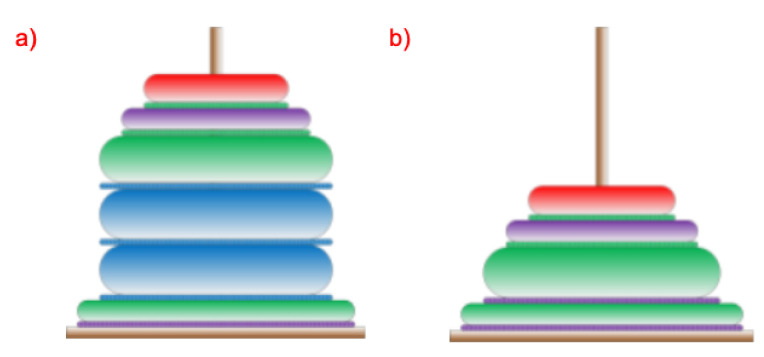
Sample Towers of Hanoi and their corresponding descriptions: (**a**) tower with one stage repeated using two interaction methods, (**b**) tower after auto-optimization: suggesting the green block as the first/default interaction method.

Each time a process is completed, a tower specific to the process is created. In terms of tower appearance analysis, the following can be distinguished: the average number of repetitions of queries carried out by the system user during the process and the occurrence of multiple queries for individual stages. Too many repetitions of the same or similar queries may indicate the inefficiency of the information delivery. In the case of multiple queries, in a single stage, two situations can be distinguished: the first (1), in which the last stage was completed after the last multiple queries, and the second (2), in which, after repeated queries of the same nature, there was a question regarding a different form of providing information. In the first case, it can be assumed that the chosen form of information transfer is not an effective form (it needs to be improved in the system used), however, the user understood and correctly interpreted possibly all of the information provided and therefore did not want/need another form. In the second, more important case, the form of communication realized last (after which the stage has been completed) seems to be more effective than the form originally used and repeated several times. Therefore, the system wanting to perform a time optimization of the process implementation should take the last form as the default (better) form of information presentation. In general, the system should prefer solutions that minimize the time it takes to complete the stage, as the default solutions for providing information. An example is presented in Figure 5.

Of course, minimizing time cannot be the only criterion used in the process. It is also important to check the correctness of the entire process. In the case of a lightly supervised process, this check is carried out after finalizing the process. The final criterion for changing the order and form of providing information to another of shorter duration is the correct execution of the entire process. In the case of a heavily-supervised process, after recognizing the incorrect execution of a stage, it is possible to provide the user with information about its incorrect execution and ask for performing the stage again with a different communication channel, changed by the system. There is therefore local time preference but with strong feedback that allows for active, within the stage, optimization of the information transfer strategy.

An alternative way of shortening a stage completion time may be the automatic Avatar request in case the user repeats the same form of obtaining information. For example, if a user triggers a request (of the same type) three times, the Avatar can be told to ask them if they need help. Thus, it can be seen that regardless of whether the Avatar is used or not, the overall stage time is the indicator of the efficiency of a stage, and in general it can be described by a function that can be treated as a penalty function for time-inefficient solutions. When on the system side, the fact of performing a stage can be indicated by an action of the system aimed at providing information. Typical solutions include comparing competing STT providers, testing of local internet connection quality, or testing of various structures of knowledge bases.

Due to the time minimization [31,32] and the priority of the correctness of the stages, the process execution optimization function has a form dependent on the fact of the intensity of the process supervision (light vs heavy).

Let the process function $j\left(\prod_{i=1}^{n}\delta_{i};D_{k};R_{l,i};I_{m,i}\right)$ for a lightly supervised process (a general check after the process is completed) have the following form:

(1) $$\begin{array}{c} j_{n}\left(\prod_{i=1}^{n}\delta_{i};D_{k};R_{l,i\leq n};I_{m,i\leq n}\right)=T_{\max}\left(1-\prod_{i=1}^{n}\delta_{i}\right)+\\ +\sum_{i=1}^{n}T_{i}\left(D_{k};R_{l,i};I_{m,i}\right)=T_{\max}\left(1-\delta \right)+\sum_{i=1}^{n}T_{i}\left(D_{k};R_{l,i};I_{m,i}\right) \end{array}$$

and for a heavily supervised process (checked after each stage):

(2) $$\begin{array}{c} j_{i}\left(\prod_{j=1}^{i}\delta_{j};D_{k};R_{l,j\leq i};I_{m,j\leq i}\right)=T_{\max}\left(1-\prod_{j=1}^{i}\delta_{j}\right)+\\ +j_{i-1}\left(\prod_{j=1}^{i-1}\delta_{j};D_{k};R_{l,j\leq i-1};I_{m,j\leq i-1}\right)+T_{i}\left(D_{k};R_{l,i};I_{m,i}\right) \end{array}$$

with condition $j_{0}=0$

which in short can be written as:

(3) $$j_{i}(\delta_{i,j};D_{k};R_{l,j\leq i};I_{m,j\leq i})=T_{\max}\left(1-\delta_{i,j}\right)+j_{i-1}+T_{i}\left(D_{k};R_{l,i};I_{m,i}\right),$$

where:

$T_{max}$—the maximal acceptable process time,
$\delta_{i}$ or $\delta_{j}$—Kronecker delta specifying the correct or incorrect execution of a process step,
$D_{k}$—factor determining employee experience,
$R_{l,i}$ or $R_{l,j}$—element of the set of possible actions of the system user,
$I_{l,i}$ or $I_{l,j}$—part of a set of possible system actions when providing information,
$T_{i}$—changes in execution time of a stage and a process.

Then the optimization task performed by the system, to minimize the time of execution while meeting the conditions of the correctness of the process, in the case of the lightly supervised process has the following form:

(4) $$\begin{array}{c} \mathrm{Min}\;j_{n}\left(\delta_{i\leq n};D_{k};R_{l,i\leq n};I_{m,i\leq n}\right)\\ \forall R_{l,i\leq n}\in \left\{W_{i\leq n}\right\}\cup W_{D,i\leq n}\left(D_{k}\right)\\ \forall I_{m,i\leq n}\in \left\{P_{i\leq n}\right\}\cup P_{D,i\leq n}\left(D_{k}\right) \end{array}$$

and in the case of a heavily supervised process:

(5) $$\begin{array}{c} \mathrm{Min}\;j_{i}\left(\delta_{i,j\leq i};D_{k};R_{l,j\leq i};I_{m,j\leq i}\right)\\ \forall R_{l,j\leq i}\in \left\{W_{j\leq i}\right\}\cup W_{D,j\leq i}\left(D_{k}\right)\\ \forall I_{m,j\leq i}\in \left\{P_{j\leq i}\right\}\cup P_{D,j\leq i}\left(D_{k}\right) \end{array}$$

where:

$\left\{W_{i\leq n}\right\}$ or $\left\{W_{j\leq i}\right\}$—set of possible system user actions,
$W_{D,i\leq n}\left(D_{k}\right)$ or $W_{D,j\leq i}\left(D_{k}\right)$—default action when no action is taken by a user,
$\left\{P_{i\leq n}\right\}$ or $\left\{P_{j\leq i}\right\}$—set of possible system responses,
$P_{D,i\leq n}\left(D_{k}\right)$ or $P_{D,j\leq i}\left(D_{k}\right)$—set of system default responses.

It is noteworthy that the value of the $j_{i}$ function, after *i* reaches *n* is equal to $j_{n}$.

The above functions allow optimization of system operations in both heavily and lightly supervised processes.

## 3. Results

The HINT system was tested in many environments, including a window assembly line and a reception desk. Although the stages of these processes are naturally differently defined, due to the versatility of the HINT system it was possible to apply them in a common programming and hardware space. Below are the results from the implementation research, where the mechanism of stage assessment and its usefulness proposed in Section 2.7 was used in three areas: optimization of process stages, automatic detection of the need to attach an Avatar, detection of events that may affect UX.

The process of window assembly was analyzed at the stand supervised by the HINT system. This process was divided into 20 stages and was carried out in the “lightly supervised” mode, with optional Avatar support. The process was repeated in two rounds: before and after optimization, and the courses of individual stages were recorded. In addition, each user completed process assessment forms, which are the basis of the UX test, and at the end of the process the window assembly quality was assessed.

**Figure 6:**
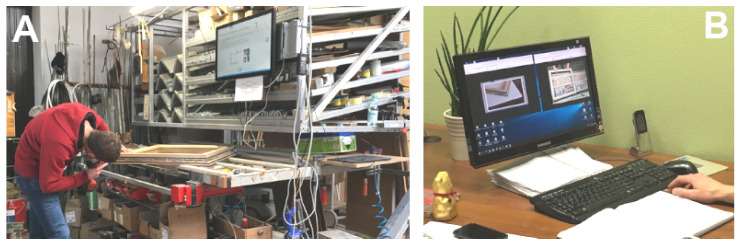
One of the testbed implementations for the proposed idea: (**A**) window assembly stand equipped with the speech- and touch-controlled HINT system, and (**B**) remote Avatar console.

The process of window assembly was analyzed at the stand (see Figure 6A) supervised by the HINT system. This process was divided into 20 stages and was carried out in the “lightly supervised” mode, with optional Avatar support (Figure 6B). The process was repeated in two versions: before and after optimization, and the employees were untrained for each attempt. The courses of individual stages were recorded. In addition, each user filled in process assessment forms, which are the basis of the UX test, and after completing the process, the window assembly quality was evaluated.

Volunteers (“employees”) were randomly selected from a group of people with no experience in window assembly. Their only support and “hint” was the HINT system, offering a touch screen with text hints, photos and videos for each stage of assembly. Every employee had the ability to interact using voice commands and touchscreen. In case of a problematic situation, employees had the possibility to connect to the expert/Avatar (video or voice only). The entire assembly process was independently monitored. In the first phase of tests (before optimization), it was the employee who independently decided if and when he would use the possibility of contacting the Avatar. In the second phase of testing (after optimization), the HINT system used the User Experience Sensor and with its help, it identified the need to attach an Avatar. The comparison of stages are presented in Table 1.

Detection of problematic stages enabled undertaking optimization actions, in the area of access to information form, most commonly used in a given stage. After the first series of 10 procedural performances, two stages were identified, due to which the Hanoi model was significantly different from the expected one. These were stages 2 (assembly of the gasket) and 6 (assembly of a two-part fitting element). These stages exceeded the time limit several times during which employees used many variants of obtaining information (description, photos and diagrams, videos) and made the most calls to an Avatar. After optimizing the information flow in stages 2 and 6, 10 subsequent process cycles were performed. Within the analyzed groups, all employees were different, and had similar competences in operating the HINT system.

Selected quantities describing the 20-stage window assembly process were compared in two groups of employees supported by the HINT system (see Table 1): (1) before optimization and (2) after optimization based on the analogy with the Towers of Hanoi.

After optimization, the correlation of the signal from the analysis of a stage (indicating a stage with a problem) with the instance of initiating an Avatar session by the employee was 0.8. The analysis of UX surveys showed compliance in 82.3% with the results from the stage analysis in terms of identifying the most problematic stages.

**Figure 7:**
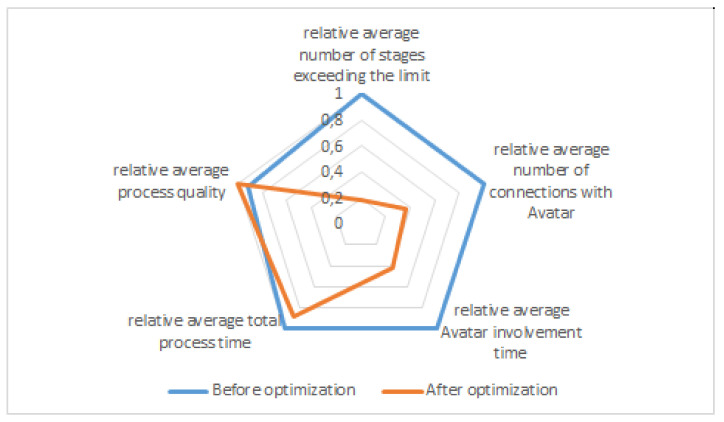
Comparison of selected quantities describing a 20-stage process of wooden window manual assembly, performed by untrained employees, assisted by the HINT system before optimization (blue), and after optimization, using stage analysis based on an analogy to the Tower of Hanoi (orange).

In the radar chart (Figure 7), it was assumed that the maximum values from Table 1 will have a relative measure equal to 1.

Basing on this graph, it can be concluded that in the result of system training the number of exceedances of the maximum time allocated to perform a given stage has mostly decreased. Therefore, the time required to complete a given stage has been normalized in relation to the maximum time allocated for its performance. After the learning, the workload of the Avatar and the average number of Avatar calls made by an employee (or the system supervising the execution of stages) also decreased significantly. Moreover, the total time to complete a given process and the quality have improved.

## 4. Discussion

The authors searched for a sensor enabling diagnostics and evaluation of processes performed in subsystems during the interaction of the AI system (with the possibility of optional support of a human-expert) with an employee.

The main challenges involved designing a sensor capable of:

- detecting the bottlenecks in the operation of subsystems that would cause a loss of fluidity of interaction, and
- detecting the moment when a human-expert resource should be attached automatically (as a result of the AI system’s decision), most likely resulting from a local inefficiency and lack of process optimization of a current stage of the process.

### 4.1. Comparison with Other Concepts

One of the key system features, as opposed to other concepts, is that a man–machine interaction system that includes the proposed User Experience Sensor, becomes a self-optimizing interaction system. Integrating a human expert into the information path, (1) on demand or (2) in the case of uncertainty, gives the possibility to leverage the quality of communication (thus user experience) to a better level.

Unlike popular interaction systems and platforms (which are usually an implementation of a Chatbot), where the system/platform uses one particular form of communication (text/voice/data), the presented HINT system includes multiple forms of information transfer (and interaction) between the system and the user. This raises an opportunity for the cooperation between the system and man, while also making it more efficient.

The presented system is being used for the analysis of the effectiveness of forms of information transfer. The target place of application of the system is the production plant in which the system is to be used for training new employees as well as to support the production process, to leverage the performance of individual employees within every stage of the production process.

The operation of the optimization algorithm allows us to increase the efficiency of information transfer within the process, which is the result of shortening the time needed by an employee to understand the requirements of particular process stages.

Moreover, as a result of optimization, it is possible to change the order in which subsequent stages are carried out even if this results in a significant shortening of the overall production time. This allows for the possibility of applying a local change within the default order of stages.

As a result of (1) the system’s participation in the process of providing and retrieving information regarding each process stage, (2) repetitive use of optimization and (3) the possibility of using the Avatar system to improve the operation of the system, it is possible to determine the default order of messages within information path and the default forms of information, allowing for a reduction of the overall process time.

### 4.2. Method Evaluation and Discussion

The conducted research confirms that the use of a system containing a module that enables process optimization allows us to shorten the process execution time while ensuring its correctness.

The mechanism proposed by the authors makes it possible to detect and identify disturbances in interaction, and thus makes it possible to take actions to improve the system in subsequent actions or to activate/attach a human-expert earlier than it would result from the conscious action of the interlocutor. The observations of the implementation within the HINT system show that, in problematic situations, users choose to use the possibility to connect with the human expert as the very last option, often unduly delaying this possibility in time, wasting the processing time. The proposed (and implemented within the HINT system) automatic mechanism for sensing the time pattern deviation and/or interaction form preferences deviation (calculated in the previous interaction iterations of the process, for a particular interlocutor) significantly reduces the time for human-expert intervention, as well as the overall process time.

### 4.3. Future Work

One of the challenges when designing systems that assist or replace humans in production processes is the insufficient availability of qualified employees resulting from the changes in the production processes for which the system was prepared, or unacceptable cost and resources needed for the adaptation of the production processes.

Taking into account the functionality of the proposed User Experience Sensor (designed as a module for assessing the interaction flow, and used for optimization of process stage efficiency and correctness), a promising direction of exploration and research seems to be the broadly understood adaptation mechanisms of AI-controlled systems.

## 5. Conclusions

In the current state of AI development, the hybrid intelligence system (HINT) understood as a combination of automatic mechanisms enabling the interaction of the knowledge system with the possibility of attaching the human factor in the form of a human-expert (Avatar) seems to be the right approach. It enables learning and optimizing the operation of systems as well as professional activation of groups of people who have been less active so far, e.g., due to disabilities. Given the remote nature of the work, these types of systems reduce the geographical limitations associated with the local availability of experts.

The method proposed by the authors to control the process supervised by a hybrid intelligence system for stage time optimization, as well as the automatic detection of the need to initialize the Avatar session, were implemented in a prototype. Although these conceptions were not investigated widely in literature and neither was their impact on the UX, the first real-life implementation tests in the prototype installation give promising results. At this stage, the main challenge of the future work will be parameterizing and benchmarking the UX of the proposed conception.

## Figures and Tables

**Table 1:** Comparison of selected quantities describing a 20-stage process of wooden window manual assembly, performed by two employee groups, assisted by the HINT system: before and after optimization using stage analysis based on an analogy with the Towers of Hanoi.

	Average Number of Stages Exceeding the Limit	Average Number of Connections with Avatar	Average Avatar Involvement Time [s]	Average Total Process Time [min]	Average Process Quality (0–100%)
Before Optimization	3.2	1.9	62 s	52	66
After Optimization	0.6	0.7	26 s	46	72

## Abbreviations

The following abbreviations are used in this manuscript:

HINT	Hybrid Intelligence System;
UX	User Experience;
HCI	Human–Computer Interaction;
HMI	Human–Machine Interface;
STT	Speech to Text;
NLP	Natural Language Processing;
NLU	Natural Language Understanding;
VoIP	Voice over IP.
